# The Sycosis of Hahnemann

**Published:** 1890-07

**Authors:** A. McNeil

**Affiliations:** San Francisco, Cal.


### THE SYCOSIS OF HAHNEMANN.

A. McNeil, M. D., San Francisco, Cal.

Hahnemann estimated that seven-eighths of all chronic diseases arise from psora, and the other eighth from syphilis and sycosis. It is evident that the far greater part are from the former, so that diseases arising from suppressed fig-warts must be rare. Most of the observing physicians, I venture to say, are of the same opinion. Owing to this rarity we seldom have the opportunity of verifying his views as applied to sycosis, so that I consider it may be instructive if I report a case which occurred to me.

February 17th, 1890, I was called to see Mr. ——. He is about fifty-five, has always been delicate and has had several attacks like the present but less severe. He served in the army during the war, but was discharged on account of chronic diarrhœa, and since then his bowels have always been slightly inclined to looseness. Has had asthma occasionally or, more strictly, in certain locations, since boyhood.

He called me on account of a hemorrhage from the glans penis. I found what looked like a small abrasion on the upper portion near the corona. From the description he gave of its first appearance, I pronounced it herpes preputialis. The hemorrhage had been very profuse although it came from so insignificant looking lesion.

I gave Sulph.$^{cc}$, which appeared to materially lessen the bleeding, but did not stop it. I made several changes, giving also Apis, Rhus, and other remedies until April 3d. There has been no material benefit, and in addition to the abrasion a glandular swelling in the left inguinal region had appeared which had now reached the size and shape, as my patient expressed it, of a mouse. I told him that there was something about his case which I had not learned, and proceeded to examine him *de novo*. He said that before the war he had had warts (condylomata) on his penis. He had no appetite and an extreme aversion to meat, the pain in the glans at the point affected was as if there was a splinter in the sore.

On these symptoms and his history I administered Nitric acid$^{500}$ (the antisycotic that ranks second only to Thuja) in water, to take four teaspoonfuls. Then to be followed by placebos. Improvement set in immediately and proceeded rapidly without any repetition or change of the remedy. In three weeks he was well barring weakness.

I forgot to say that the fig-warts had been removed by instruments. I firmly believe that the sycotic miasm or virus had been deteriorating his health all of those thirty years, and by the indicated remedy was so soon rendered innocuous. Hahnemann's services to mankind consist in other things besides the discovery and formulation of the law of the similars. The psoric theory was a discovery of transcendental value, and notwithstanding many of his professed followers have discarded it, the more advanced minds of the old school are taking it up and ultilizing it by forbidding the suppression of disease manifestations by external applications.

